# HIV screening and retention in care in people who use drugs in Madrid, Spain: a prospective study

**DOI:** 10.1186/s40249-021-00894-5

**Published:** 2021-08-19

**Authors:** Pablo Ryan, Jorge Valencia, Guillermo Cuevas, Jesús Troya, Juan Torres-Macho, María José Muñoz-Gómez, Nuria Muñoz-Rivas, Isabel Canorea, Sonia Vázquez-Morón, Salvador Resino

**Affiliations:** 1 Hospital Universitario Infanta Leonor, Madrid, Spain; 2 Universidad Complutense de Madrid (UCM), Madrid, Spain; 3 Instituto de Investigación Sanitaria Gregorio Marañón (IiSGM), Madrid, Spain; 4 Unidad de Reducción de Daños “SMASD”, Madrid, Spain; 5 Unidad de Infección Viral e Inmunidad, Centro Nacional de Microbiología, Instituto de Salud Carlos III, Carretera Majadahonda- Pozuelo, Km 2.2, 28220 Majadahonda, Madrid Spain

**Keywords:** HIV, Point-of-care, Screening, People who use drugs, Retention in care, Hepatitis C, Dried blood spot, Antiviral treatment

## Abstract

**Background:**

The burden of human immunodeficiency virus (HIV) infection in people who use drugs (PWUD) is significant. We aimed to screen HIV infection among PWUD and describe their retention in HIV care. Besides, we also screen for hepatitis C virus (HCV) infection among HIV-seropositive PWUD and describe their linkage to care.

**Methods:**

We conducted a prospective study in 529 PWUD who visited the “Cañada Real Galiana” (Madrid, Spain). The study period was from June 1, 2017, to May 31, 2018. HIV diagnosis was performed with a rapid antibody screening test at the point-of-care (POC) and HCV diagnosis with immunoassay and PCR tests on dried blood spot (DBS) in a central laboratory. Positive PWUD were referred to the hospital. We used the Chi-square or Fisher’s exact tests, as appropriate, to compare rates between groups.

**Results:**

Thirty-five (6.6%) participants were positive HIV antibodies, but 34 reported previous HIV diagnoses, and 27 (76%) had prior antiretroviral therapy. Among patients with a positive HIV antibody test, we also found a higher prevalence of homeless (*P* < 0.001) and injection drug use (PWID) (*P* < 0.001), and more decades of drug use (*P* = 0.002). All participants received HIV test results at the POC. Of the 35 HIV positives, 28 (80%) were retained in HIV medical care at the end of the HIV screening study (2018), and only 22 (62.9%) at the end of 2020. Moreover, 12/35 (34.3%) were positive for the HCV RNA test. Of the latter, 10/12 (83.3%) were contacted to deliver the HCV results test (delivery time of 19 days), 5/12 (41.7%) had an appointment and were attended at the hospital and started HCV therapy, and only 4/12 (33.3%) cleared HCV.

**Conclusions:**

We found almost no new HIV-infected PWUD, but their cascade of HIV care was low and remains a challenge in this population at risk. The high frequency of active hepatitis C in HIV-infected PWUD reflects the need for HCV screening and reinforcing the link to care.

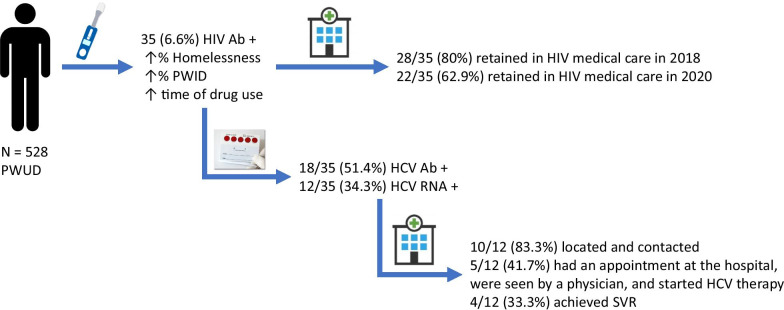

## Background

Human immunodeficiency virus (HIV) infection in people who use drugs (PWUD), such as cocaine and heroin, is a significant global issue, particularly among people who have injected drugs (PWID) [1]. The global HIV prevalence in PWID is 17.8%, and around 50% are hepatitis C virus (HCV) antibody-positive [2]. Despite this, the incidence of new HIV and HCV diagnoses among PWID has decreased sharply since 2010 due to the implementation of harm reduction services and treatment as prevention strategies [3–6]. However, the level of coverage of these strategies is different and varies among regions and remains low globally [7].

PWUD are exposed to multiple adverse environments that can increase the risk of HIV and HCV acquisition [8, 9]. The most determining factors in increasing the risk of HIV and HCV acquisition are intravenous drug use, increased frequency of injection, syringe sharing, homelessness, incarceration, and exposure to physical and sexual violence, among others [8–12]. Both HIV and HCV infections are usually diagnosed at hospitals using standardized tests, but this causes very high tracking losses among PWUD. Strategies to scale up HIV and HCV testing, such as rapid tests at the point of care (POC) and the use of mobile medical units, increase the probability of diagnosis among PWUD [13, 14]. Another alternative is the use of dried blood samples (DBS) and to carry out the diagnosis in reference centers. However, this strategy has limitations, such as the diagnosis is delayed and hinder the link with medical care [15, 16].

The HIV continuum of care may be used as a public health tool to measure the effectiveness of health systems and monitor progress towards the goals set to end the HIV epidemic, consisting of several steps required to achieve HIV suppression [17, 18]. However, different definitions of the HIV continuum of care are used according to countries, and there are disparities between population subgroups [19]. Moreover, linkage and retention in care after HIV diagnosis present unique challenges for PWUD infected with HIV [13, 20]. The most limiting factors related to low HIV retention in care are the mistrust between providers and PWUD, intravenous drug use, binge drinking, younger age, ethnic minorities, HCV coinfection, psychiatric illness, lack of insurance, male sex, and low education level [13, 21]. Besides, both HIV and HCV therapies can only be prescribed at specialized health centers, where PWUD do not usually go, making the treatment still inadequate [13, 14]. Therefore, holistic services are essential to HIV retention in care for PWUD, including health insurance, counseling, adherence support, mental health, substance abuse treatment, and housing assistance [17]. Data related to people retained in HIV care in PWUD attended in harm reduction services remain scarce. We aimed to screen HIV infection among PWUD and describe their retention in HIV care. Besides, we also screen for HCV infection among HIV-seropositive PWUD and describe their linkage to care.

## Methods

### Study design

We conducted a prospective study in 529 PWUD in the last year and who visited the “Cañada Real Galiana” (Madrid, Spain), a shantytown where much of the illicit drugs of the region are sold and consumed. Around 4000–6000 people a day get to this place to obtain drugs, and a rapid HIV test was offered to everyone by order of appearance (consecutive sampling). They were invited to a screening of blood-borne infections, and all HIV tests were performed before the interview. The study period was from June 1, 2017, to May 31, 2018, when the time limit established in the research project was reached. The inclusion criteria were: (1) age ≥ 18 years; (2) PWUD; (3) ability to sign informed consent; (4) provide contact information for its subsequent location in case of having a positive result. This cohort has previously described in a study about HCV screening [22].

In the HIV screening phase (baseline), a mobile unit consisting of a van adapted for the project and a satellite car was used. In this mobile unit, a trained nurse and a navigator/educator performed HIV screening using the OraQuick ADVANCE Rapid HIV-1/2 Antibody Test (OraSure Technologies, Bethlehem, PA, USA). The sensitivity and specificity of this HIV antibody test is 98% and 100%, respectively [23]. The test results were returned in 20 min; time used to collect epidemiological data, and recommend prevention and harm reduction. Moreover, we collected fingerstick DBS using Whatman cards and sent them to the Instituto de Salud Carlos III (ISCIII) to determine HCV antibodies and HCV RNA. In this case, the HCV screening results were given to the participant 1 to 2 weeks later.

In the linkage and retention in care phase (follow-up), participants with a rapid HIV serological test were offered to be referred to the hospital the same day, both to participants with a new HIV diagnosis and those known diagnoses. In Spain, HIV treatment is only prescribed by a physician at hospitals. HIV-infected patients were transported to the hospital by car, and the navigator accompanied the patient during the process of initiation or re-initiation of antiretroviral therapy (ART). Most of the patients were transported to the Fast-Track Clinic at “Infanta Leonor” Hospital, due to the geographic proximity, where patients were examined and treated against HIV infection. Retention in care was measured at two points in time (2018 and 2020) by asking patients whether they had an appointment at the HIV clinic and received ART in the previous year. This information was further confirmed by consulting the medical records.

Moreover, HIV-infected patients with active hepatitis C were also contacted and referred to the hospital, but several days later, where were examined and treated against HCV infection. In this case, the navigator also accompanied the patient to the hospital to initiate HCV treatment and to confirm the cure of hepatitis C, that is, to achieve a sustained virological response (SVR) defined as undetectable serum HCV RNA 12 weeks after completing antiviral treatment with direct antiviral agents. When a patient refused to be referred to the hospital that day, he was subsequently contacted to try referral another day.

### Data management

Epidemiological data (sex, age, nationality, income, and homeless status), substance abuse [time of drug use, type of drug used, the current route of drug administration, daily alcohol intake, benzodiazepine, and injecting drug users (IDUs)], healthcare (regular medical care, health insurance, previous anti-HIV testing, opioid substitution therapy, and psychiatric treatment), and sexual risk behavior (stable partner, men who have sex with men and commercial sex work) in the past 12 months were collected by researchers at the POC by an online form on a mobile device (the participant did not self-complete any form/information) using the Research Electronic Data Capture (REDCap, Vanderbilt University, Nashville, TN, USA) [24], hosted at the Asociación Ideas for Health. Clinical data related to the diagnosis and treatment of HIV infection were collected from hospital medical records.

The homeless lived on the street or in a homeless shelter. The alcohol intake (> 50 g/day) was self-reported. PWUD were those who had ever used drugs in the last year. PWID were those who ever used intravenous drugs in the previous year.

### Laboratory assays

DBS samples were obtained by fingerstick using Whatman 903 cards, dried for four hours at room temperature (~ 25 °C), and subsequently stored at 4 °C in individual plastic bags with a desiccant and zipped closed. Then, within 15 days of collection, samples were sent refrigerated (~ 4 °C) to the ISCIII and stored at − 80 °C until processing. We processed DBS samples according to the described protocol [25]. Briefly, two discs from the Whatman 903 cards were added to a solution with 0.05% Tween in PBS at 25 °C. After, samples were centrifuged at 1200×*g* for 30 min and incubated overnight at 4 °C. On the morrow, DBS eluates were stored at − 80 °C until analysis.

DBS eluates were tested for HIV and HCV. We evaluated anti-HIV antibodies (Murex HIV Ag/Ab Combination Kit, DiaSorin, Saluggia, Italy), anti-HCV antibodies (Murex anti-HCV kit, v. 4.0, DiaSorin, Saluggia, Italy) in the eluates using an ETI-Max 3000 instrument (DiaSorin, Saluggia, Italy). According to a previously described protocol [25], the diagnosis of active hepatitis C was performed only in people with positive HCV antibodies. HCV RNA was extracted using the mini-kit DSP Virus/Pathogen (Qiagen, Hilden, Germany) and was detected using a qualitative SYBR Green RT-PCR assay, with a limit of detection of 960 IU/ml.

### Outcome variables

We analyzed a series of outcome variables related to (1) HIV: HIV infection (positive OraQuick rapid test) and HIV retention in care (having an appointment at the hospital and receiving HIV therapy). (2) Hepatitis C: HCV infection (positive anti-HCV test and positive HCV-RNA test) and HCV linkage to care [delivering of HCV test results; having an appointment at the hospital; being seen by a physician once at the hospital; starting HCV therapy, and achieving sustained virological response (SVR)].

### Statistical analysis

Statistical analysis of the population description was performed using the Mann–Whitney tests for continuous variables and the Chi-square or Fisher’s exact tests for categorical variables, as appropriate. Besides, the rates of patients infected with HIV and HCV and patient retention in HIV care were compared between groups using Chi-square or Fisher’s exact tests. We also analyze the association between the significant characteristics of the patients (see Table 1) and a positive result in the HIV test by multivariate logistic regression.

**Table 1:** Baseline epidemiological characteristics of the population screened

Characteristics	All	HIV (−)	HIV (+)	*P*-value
*n*	529	494	35	
Age (years)	42 (35; 48)	42 (34; 48)	48 (41; 50)	0.003
< 30	47 (8.8%)	47 (9.5%)	0 (0.0%)	n.s
30–40	161 (30.4%)	154 (31.2%)	7 (20.0%)	n.s
40–50	216 (40.8%)	198 (40.1%)	18 (51.4%)	n.s
≥ 50	105 (19.8%)	95 (19.2%)	10 (28.6%)	n.s
Male	420 (79.4%)	392 (79.3%)	28 (80.0%)	n.s
Origin				
Spain	419 (79.2%)	392 (79.3%)	27 (77.1%)	n.s
Eastern Europe	55 (10.4%)	49 (9.9%)	6 (17.1%)	n.s
Western Europe	17 (3.2%)	16 (3.2%)	1 (2.8%)	n.s
North Africa	23 (4.3%)	22 (4.4%)	1 (2.8%)	n.s
America	7 (1.3%)	7 (1.4%)	0 (0.0%)	n.s
Other	8 (1.5%)	8 (1.6%)	0 (0.0%)	n.s
Homeless	105 (19.9%)	90 (18.3%)	15 (42.8%)	< 0.001
Drugs use				
Decades of drug use (*n* = 489)				
< 1 year	6 (1.2%)	6 (1.3%)	0 (0.0%)	n.s
1–9 years	83 (17.0%)	82 (18.0%)	1 (2.9%)	0.024
10–19 years	171 (35.0%)	165 (36.3%)	6 (17.6%)	0.028
20–29 years	134 (27.4%)	120 (26.4%)	14 (41.2%)	n.s
≥ 30 years	95 (19.4%)	82 (18.0%)	13 (38.2%)	0.004
Type of drug used				
Heroin	412 (78.0%)	383 (77.5%)	29 (82.9%)	n.s
Cocaine	476 (90.0%)	443 (90.0%)	33 (94.3%)	n.s
Marihuana	71 (13.4%)	69 (14.0%)	2 (5.7%)	n.s
Alcohol (> 50 g/day)	50 (9.4%)	49 (9.9%)	1 (2.8%)	n.s
Go for drugs daily				
The current route of drug administration				
Injected	177 (33.4%)	149 (30.2%)	28 (80.0%)	< 0.001
Smoked	432 (81.6%)	404 (81.8%)	28 (80.0%)	n.s
Snorted	135 (25.5%)	130 (26.3%)	5 (14.3%)	n.s
Benzodiazepines prescribed	39 (7.4%)	35 (7.1%)	4 (11.4%)	n.s
Had injected drugs (IDUs)	254 (49.7%)	223 (46.8%)	31 (88.6%)	< 0.001
Shared syringes in the previous year	21 (8.5%)	17 (7.9%)	4 (13.3%)	n.s
Shared paraphernalia in the previous year	90 (40.4%)	89 (40.6%)	1 (25.0%)	n.s
IDUs active in the previous year	127 (26.6%)	118 (26.6%)	9 (26.5%)	n.s
Healthcare				
No health insurance	74 (15.4%)	67 (14.9%)	7 (21.9%)	n.s
Primary care assistance	138 (26.1%)	134 (24.1%)	4 (11.4%)	0.041
Never tested for HIV	57 (11.6%)	57 (11.6%)	0 (0.0%)	n.s
Last HIV test > 1 year	124 (34.8%)	122 (34.7%)	2 (50.0%)	n.s
Opioid substitution therapy	142 (27.7%)	123 (25.8%)	19 (54.3%)	< 0.001
On psychiatric treatment	148 (28.9%)	134 (28.1%)	14 (41.2%)	n.s
HCV-related sexual risk behavior				
Stable partner	204 (46.3%)	188 (45.6%)	16 (55.2%)	n.s
Sexual risk behavior in last year	57 (19.1%)	56 (20.3%)	1 (4.5%)	n.s
Men who have sex with men	9 (2.7%)	7 (2.3%)	2 (8.3%)	n.s
Commercial sex work (only women)	34 (9.6%)	31 (9.4%)	3 (12.5%)	n.s

Statistics: Values are expressed as number (percentage) and median (interquartile range)

*HCV* hepatitis C virus; *IDU* injection drug user; *P-value* level of significance; *n.s.* not significant

All statistical analyses were performed using IBM SPSS v25 (IBM Corp, Armonk, NY, USA). Figures were created using GraphPad Prism v8.0 (GraphPad Software, Inc., San Diego, CA, USA). All *P*-values were two-tailed. Statistical significance was considered with *P* < 0.05.

## Results

### Characteristics of the study population

Overall, the population had an age of 42 years, 79% were male, 20% were homeless, and 21% were migrants (Table 1). Regarding drug use, 46.8% had used drugs for more than 20 years, heroin and cocaine were the most used drugs, and around 50% had previously injected drugs. Regarding healthcare, 88% of participants had usual care in a primary health center, 11% were never tested for HIV infection, 35% had not been tested for HIV infection in the last year, and 28% had taken opioid substitution therapy. Concerning sexual risk behaviors, 46% of participants had a stable partner, and 19% had sexual behavior at risk of HIV infection.

### HIV screening and linkage to care

We found 35/529 participants with positive HIV antibodies by rapid HIV test [6.6%, 95% confidence interval (*CI*) 4.5–8.7%)], which were confirmed by a serological HIV assay performed on DBS samples (100% of concordance). All participants received their HIV test results at the POC, and they were offered the support of an educator/navigator and specialized medical care at the hospital. Among the 35 individuals with positive HIV antibodies, 34 reported a previous diagnosis of HIV infection, and 27 (76%) reported taking ART. Of the 35 HIV-positive patients, 28 (80%) were retained in HIV medical care in 2018 and only 22 (62.9%) in 2020. No significant differences were found when the population was stratified by homeless, PWID, and decades of drug use (*P* > 0.05).

### Factors associated with a positive result in the HIV test

HIV-infected patients were older, more often homeless, long-term drug users, injecting was the main drug administration route, and were on opioid substitution therapy, but they do not usually go to their primary care center (Table 1).

**Fig. 1:**
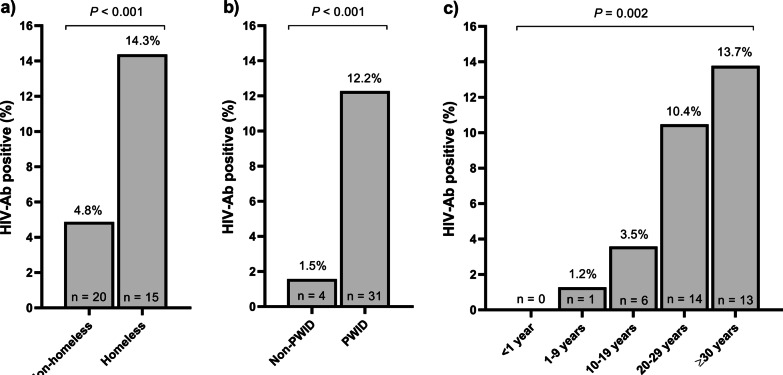
Prevalence of HIV infection in people who use drugs (PWUD) from Cañada Real Galiana. **a** Population stratified by homeless; **b** population stratified by injection drug users; **c** population stratified by decades of drug use. *PWID* people who injected drugs; *HIV* human immunodeficiency virus; *HIV-Ab* antibodies against HIV; *P*-value, level of significance

We also performed a multivariate logistic regression for these significant variables, finding that only three factors were independently related to HIV infection (Table 2): homelessness [adjusted odds ratio (a*OR*)  3.16; *P* = 0.003], PWID (a*OR* 5.95; *P* = 0.001), and decades of drug use (a*OR* 1.98; *P* = 0.001). Among patients with a positive HIV antibody test, we also found a higher prevalence of homeless (*P* < 0.001; Fig. 1A) and injection drug use (PWID) (*P* < 0.001; Fig. 1B), and more decades of drug use (*P* = 0.002; Fig. 1C).

**Table 2:** Sociodemographic and epidemiological characteristics associated with HIV infection among people who use drugs

Variables	Univariate		Multivariate	
	*OR* (95% *CI*)	*P*-value	a*OR* (95% *CI*)	*P*-value
Age (years)*	1.05 (1.01–1.09)	0.007	–	–
Homeless	3.34 (1.64–6.78)	< 0.001	2.64 (1.23–5.68)	0.013
Injection drug user	9.42 (3.27–27.09)	< 0.001	5.37 (1.81–15.93)	0.002
Time using drugs (decades)	1.25 (1.09–1.43)	< 0.001	1.22 (1.05–1.42)	0.008
Primary care assistance	0.34 (0.12–1.01)	0.050	0.56 (0.18–1.73)	0.321
Opioid substitution therapy	3.42 (1.71–6.85)	< 0.001	1.95 (0.91–4.16)	0.081

Statistical analysis: The association analysis was performed using logistic regression. (*), age was discarded in multivariate analysis because it was highly correlated with time using drugs (*r* = 0.601; *P* < 0.001)

*OR* odds ratio; *aOR* adjusted odds ratio; 95% *CI* 95% confidence interval

### HCV screening and linkage to care in HIV-infected people

**Fig. 2:**
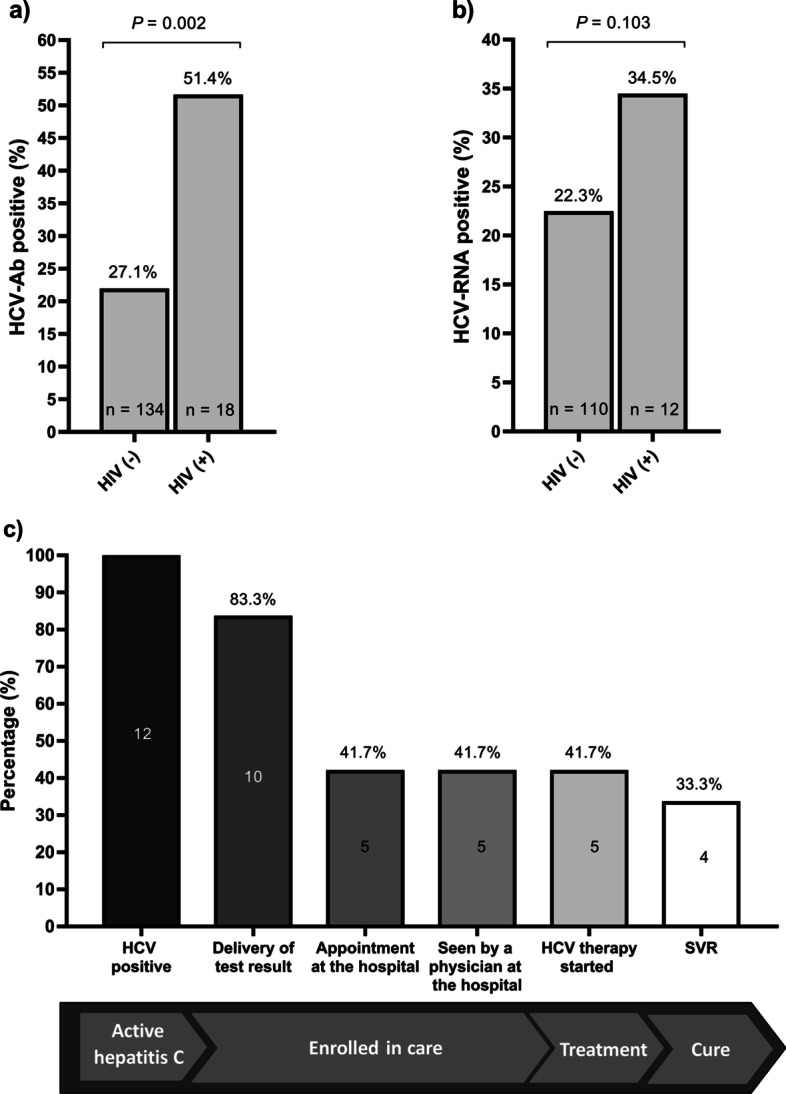
Prevalence of hepatitis C infection (**a**) and linkage to care (**b**) among HIV-infected drug users in Cañada Real Galiana. *HIV* human immunodeficiency virus; *HCV* hepatitis C virus; *HCV-Ab* antibodies against HCV; *HCV-RNA* HCV ribonucleic acid; *SVR* sustained virological response; *P-value* level of significance

The prevalence of HCV antibodies was higher in HIV-infected subjects than in HIV uninfected participants (Fig. 2A; 51.4% vs 27.1%; *P* = 0.002). Furthermore, the prevalence of active hepatitis C (HCV RNA test positive) was 34.3% in HIV patients and 22.3% in non-HIV participants (Fig. 2B; *P* = 0.103). Of the 12 HIV-infected subjects with active HCV infection, 10 (83.3%) were located and contacted to deliver the HCV test results (median delivery time of 19 days (interquartile range = 14–25)). Of these, 5 (41.7%) had an appointment at the hospital, were seen by a physician, and subsequently started HCV therapy. Finally, only 4 (33.3%) patients achieved SVR, 75% of those who began HCV therapy (Fig. 2C). Overall, we did not find significant differences in the linkage to care between HIV-uninfected and HIV-infected patients (*P* > 0.05).

## Discussion

We performed a prospective observational study in PWUD from a shantytown in Madrid to estimate the HIV seroprevalence and the HCV/HIV coinfection proportion among PWUD. Furthermore, the effectiveness of referral to a specialized clinical care unit was also evaluated. This strategy involved using a mobile unit to search and reach potential participants, POC testing, and a navigator to refer and accompany the PWUD to the hospital. Our most relevant findings were the following: (i) HIV seroprevalence was 6.6%, higher in homeless, PWID, and long-term drug users; and the vast majority of HIV-infected PWUD already knew their HIV infection, and 75% reported to be on ART; (ii) retention in care among HIV-infected PWUD was low during the study period (80%) and two years after completing the HIV screening study (62.9%); (iii) one-third of HIV-infected PWUD were coinfected with HCV, and of these, only a third started treatment and were cured.

As is widely known, HIV transmission is high among PWID due to risky behaviors, such as unsafe injections. Homelessness in itself is also a risk factor for HIV infection [26]. This could be explained because independent risk factors, such as injection drug use, engaging in unsafe injection drug‐use practices, mental health disorders, and a history of incarceration, are common in this population [27, 28]. Our study is in accordance with previous estimates of HIV prevalence among PWUD in Western and Central Europe [29], and confirms the higher HIV prevalence in homeless PWUD screened and people with a longer drug injection duration. In the last years, growing evidence has shown the harmful effect of housing instability on the health and social outcomes of PWID, including an elevated risk of HIV and HCV acquisition [30]. Indeed, homeless PWID often faces profound disadvantage and have multiple competing needs [31]. Therefore, a comprehensive approach that provides housing and addresses many of the interlinked health and social concerns of this population is necessary to reduce HIV risk in this population.

Long-term retention in HIV care remains a challenge in vulnerable populations [32], and substance use is the most cited predictor of low retention [33]. However, other factors (e.g., medical comorbidities, addiction severity, and psychiatric disorders, among others) may interact and influence behaviors and health outcomes, such as adherence and retention of ART in care [34]. The previous step of retention in care is linkage to care, and our study was focused on the linkage to care, but there was also a follow-up period in which measures were implemented to promote retention in care. In our study, only two-thirds of the participants were retained in HIV medical care two years later, and although there is no gold standard for measuring retention in care, this value is as high as other published studies [35]. The role of navigators and the mobile units using HIV GenXpert to reinforce the linkage and retention to care in critical populations attended and screened at a POC (allowing retesting and retreatment) should be explored in further studies. In this way, expanding this focus to retention in HIV care (vs ART adherence alone) offers a more holistic point of view of HIV management and moves away from the purely medical model to account for psychosocial issues and physical comorbidities, specific to vulnerable populations, in conjunction with medication adherence. Moreover, no differences were found when the population was stratified by injection practices, homeless, and decades of drug use, possibly due to the low sample size. We think that a larger sample could demonstrate the previous findings.

We found a high prevalence of HIV/HCV coinfected patients with detectable HCV-RNA, although this group has had early access to direct-acting antiviral therapy [36], mainly because these patients were being followed in HIV clinics. While HIV specialists may continue to play a vital role in integrated and holistic care for all populations included marginalized populations [37, 38], hospital referrals are strictly necessary to start ART or hepatitis C therapy in vulnerable people who are not retained in care.

The current study has several limitations. First, the study design was observational, the sample size was small, and it was conducted in a single city, which may reduce the generalizability of our findings. Second, some data collected in the questionnaire may not be completely reliable; but this bias may be minimal because the data were collected by trained personnel. Third, some participants could have changed their residence, and therefore, no information was available on HIV linkage to care. Fourth, we assumed that patients were in retention in care by self-reports in the interview, but this information was not confirmed. Fifth, HIV viral load data were not available to corroborate if they were on effective ART and virologically suppressed. Sixth, no incentives or economic compensation were offered, so some participants may not have been included in the study. However, the educators provided food and drinks while the participants were waiting for their results. The refusal rate was no calculated.

## Conclusions

We found almost no new HIV-infected PWUD, but their cascade of HIV care was low, particularly long-term HIV retention in care, and continues to be a challenge in this population at risk. The high frequency of active hepatitis C in HIV-infected PWUD reflects the need for HCV screening and reinforcing the link to care.

## Data Availability

Datasets used and analyzed during the current study may be available from the corresponding author upon reasonable request.

## Abbreviations

a*OR*: Adjusted odds ratio
ART: Antiretroviral therapy
DBS: Fingerstick dried blood spot
HCV: Hepatitis C virus
HIV: Human immunodeficiency virus
POC: Point of care
PWID: People who have injected drugs
PWUD: People who use drugs
SVR: Sustained virological response

## Declarations

### Originality

The authors confirm that the material contained herein is entirely original.
